# Long-term three-dimensional high-resolution imaging of live unlabeled small intestinal organoids via low-coherence holotomography

**DOI:** 10.1038/s12276-024-01312-0

**Published:** 2024-10-01

**Authors:** Mahn Jae Lee, Jaehyeok Lee, Jeongmin Ha, Geon Kim, Hye-Jin Kim, Sumin Lee, Bon-Kyoung Koo, YongKeun Park

**Affiliations:** 1 Graduate School of Medical Science and Engineering, Korea Advanced Institute of Science and Technology (KAIST), Daejeon, 34141 Republic of Korea; 2 KAIST Institute for Health Science and Technology, Daejeon, 34141 Republic of Korea; 3 Tomocube Inc., Daejeon, Republic of Korea; 4 Center for Genome Engineering, Institute for Basic Science, Daejeon, 34126 Republic of Korea; 5 Department of Physics, KAIST, Daejeon, 34141 Republic of Korea; 6 Department of Life Sciences, Pohang University of Science and Technology (POSTECH), Pohang, Republic of Korea

**Keywords:** Imaging, Organogenesis

## Abstract

Organoids, which are miniature in vitro versions of organs, possess significant potential for studying human diseases and elucidating their underlying mechanisms. Live imaging techniques play a crucial role in organoid research and contribute to elucidating the complex structure and dynamic biological phenomena of organoids. However, live, unlabeled high-resolution imaging of native organoids is challenging, primarily owing to the complexities of sample handling and optical scattering inherent in three-dimensional (3D) structures. Additionally, conventional imaging methods fail to capture the real-time dynamic processes of growing organoids. In this study, we introduce low-coherence holotomography as an advanced, label-free, quantitative imaging modality designed to overcome several technical obstacles for long-term live imaging of 3D organoids. We demonstrate the efficacy of low-coherence holotomography by capturing high-resolution morphological details and dynamic activities within mouse small intestinal organoids at subcellular resolution. Moreover, our approach facilitates the distinction between viable and nonviable organoids, significantly enhancing its utility in organoid-based research. This advancement underscores the critical role of live imaging in organoid studies, offering a more comprehensive understanding of these complex systems.

## Unveiling Real-Time Morphological Details in Viable 3D Organoids

Organoids, miniature 3D structures that imitate real organs, are grown in labs to study human biology and diseases. However, their complex structures and behaviors are hard to understand due to imaging technology limitations. In this study, researchers used a method called low-coherence holotomography to study mouse small intestinal organoids. This method let them observe organoids’ growth and drug responses in real-time, without altering their natural state. They conducted an experiment involving over 120 hours of continuous imaging, providing new insights into organoid development, cell dynamics, and drug responses. The study shows that low-coherence HT can reveal detailed 3D structures and changes within organoids, such as cell division and death, with high resolution. This research could revolutionize drug development and testing and provide new insights into human biology and diseases. This summary was initially drafted using artificial intelligence, then revised and fact-checked by the author.

## Introduction

Organoids, three-dimensional (3D) multicellular structures that closely emulate organ architecture and function, are cultivated in vitro from both pluripotent and adult stem cells^1–3^. The development of organoids has transformed them into essential resources for a broad spectrum of biological research targeting organs such as the intestines^4,5^, lung^6,7^, liver^8–10^, kidneys^11,12^, and brain^13,14^. These 3D organ models surpass conventional two-dimensional (2D) cell culture methods by closely replicating the natural architecture and organization of cells found in living organisms. Organoids are formed when progenitor cells sourced from an organ’s adult stem cells or induced pluripotent stem cells undergo differentiation and organization into structures that resemble organ function and structure, mirroring the organ development process in nature. This methodology ensures that organoids retain their cellular and molecular properties more faithfully than traditional cell cultures do. By closely simulating the conditions within a living organism, organoids have become pivotal in disease modeling, facilitating the exploration of disease mechanisms and the evaluation of new drugs and therapeutic approaches^3^.

Imaging plays a crucial role in unraveling the complexity of organoids, revealing their morphology and confirming their accurate representation of in vivo counterparts. Since organoids are composed of various cell types and are 3D structures surrounded by an extracellular matrix (ECM), differentiating cell types within organoids with current imaging modalities is challenging. Thin sectioning, along with traditional staining, has been widely employed to examine tissue architecture in 2D and analyze the distribution of single and multiple stained biomarkers. Although brightfield microscopy remains instrumental for organoid visualization, it is limited in capturing their 3D complexity. Thick sectioning and imaging have been used to address the lack of 3D information available on organoids. However, thick sections impede light penetration, resulting in poor image quality and reduced resolution under light microscopy. In addition, the multiple planes of focus found in thick slices create optical clarity issues, making it difficult to distinguish between different tissue structures^15^. More advanced modalities, including confocal, multiphoton, and light-sheet microscopy, confer improved 3D imaging but have their own caveats—namely, the requisite fluorescent labeling of samples can introduce potential phototoxic effects^16,17^. Consequently, there is an ongoing quest for advanced imaging technologies that can provide high-resolution images, deep tissue penetration, and real-time observations of dynamic cellular changes in organoids without requiring time-consuming sample preparation, labeling, and imaging^18^.

In recent years, quantitative phase imaging (QPI) has gained recognition for the label-free imaging of live biological samples^19–23^. Holotomography (HT)—a 3D extension of QPI—offers a unique advantage by enabling the real-time capture of cellular dynamics in organoids without phototoxicity or photobleaching. In this work, we employ low-coherence HT for the sustained, label-free monitoring of organoids, shedding light on their developmental trajectories and pharmacological responses. Using mouse small intestinal organoids (sIOs) as our organoid imaging model system, we were able to capture 120 hours of time-lapse imaging. This approach allowed us to observe growth patterns, capture detailed subcellular structures with a lateral resolution of 155 nm and an axial resolution of 947 nm, and quantitatively evaluate organoid responsiveness to drug treatment. Low-coherence HT was uniquely effective in distinguishing viable and nonviable cells within organoids and offering unparalleled details in depicting 3D morphological shifts following drug exposure. The proposed method further enables quantitative measurements of organoid volume, protein concentration, and dry mass, setting a new standard for comprehensive and rigorous statistical assessments for biological studies of organoids.

## Materials and methods

### Organoid cultures

Mouse sIOs, derived from primary crypts isolated from the mouse small intestine, were kindly provided by the Center for Genome Engineering (Institute for Basic Science, Korea). Organoid culture media were either manually prepared or purchased. For sIO culture media preparation, advanced DMEM/F12 (Gibco, USA) was supplemented with 0.1 M HEPES (Gibco, USA), 1× Glutamax (2 mM L-alanyl-L-glutamine dipeptide) (Gibco, USA), 100 U/mL penicillin‒streptomycin (Gibco, USA), 1× serum-free B27 supplement (Gibco, USA), 1.25 mM N-acetyl-L-cysteine (NAc) (Sigma‒Aldrich, USA), 0.05 μg/mL mouse EGF (Gibco), 0.1 μg/mL human Noggin (Peprotech, USA), and 0.1 μg/mL human R-Spondin-1 (Peprotech, USA). Alternatively, the commercially available culture medium IntestiCult (STEMCELL Technologies, Canada) was prepared according to the manufacturer’s instructions. The culture medium was replaced every 2–3 days, and the organoids were passaged weekly. For passaging, both the Matrigel dome and organoids were mechanically dissociated into individual crypt domains. These individual crypts were then mixed with fresh Matrigel at a split ratio of 1:8, kept at temperatures below 4 °C, and distributed in 15 μL dome shapes onto a 48-well plate. To polymerize the Matrigel dome, the plate was placed upside-down in an incubator for 10 minutes at 37 °C with 5% CO_2_. Once the Matrigel solidified, 250 μL of fresh culture medium was added to each well.

### Imaging platform

Holotomograms of the sIOs were obtained via a low-coherence HT system (HT-X1, Tomocube Inc., Korea). The system consists of two main modules: illumination and acquisition. The illumination module features an LED light source with a center wavelength of 450 nm. A digital micromirror device is positioned in the illumination beam path to scan through multiple aperture intensity profiles, which are computationally designed to facilitate the reconstruction of the refractive index (RI).

The acquisition module is a 4-*f* imaging system equipped with a motorized objective lens and a motorized stage. The objective lens is a dry 40× lens with a numerical aperture of 0.95. The motorized objective lens scans through the axial planes of the sample, whereas motorized stage movements present different horizontal regions for measurement. The sample undergoes axial scanning over a range of 140 μm, with transmitted intensity measurements taken at intervals of 947 nm for each illumination pattern.

Subsequently, deconvolution is carried out to reconstruct the RI of the samples within the desired field of view. The low-coherence HT reconstruction involves the deconvolution of 3D intensity measurements with an optical transfer function to obtain a 3D RI image. The optical transfer function is determined by the set of aperture intensity profiles of the illumination beam; these intensity profiles are optimally designed to allow the transfer function to evenly cover the spatial frequency domain. A detailed description of the reconstruction principle can be found elsewhere^24,25^.

The low-coherence HT system provides theoretical resolutions of 155 nm and 947 nm in the lateral and axial directions, respectively. An integrated stage top incubation chamber ensures stable physiological conditions of temperature, humidity, and CO_2_ concentration during long-term imaging over several weeks. All motorized microscopic operations were controlled and monitored by the operating software TomoStudio X (Tomocube Inc., Korea).

### Sample preparation for imaging

HT-X1 is adaptable to a wide variety of commercial imaging dishes, multiwell plates, and custom imaging vessels with a bottom thickness of #1.5H. In this study, organoids were imaged via a coverslip-bottomed imaging dish (TomoDish, Tomocube Inc., Korea). Prior to imaging, the sIOs underwent mechanical dissociation during organoid passaging. The dissociated individual crypts were mixed with fresh Matrigel and then distributed as 15 μL domes onto the coverslip-bottom imaging dish. This was followed by a polymerization process. When the Matrigel solidified, it was topped with 3 mL of culture medium, and the imaging dish was positioned in the HT-X1 chamber.

For correlative fluorescent organoid imaging, a staining solution was prepared using calcein-AM (Thermo Fisher, USA) diluted 1:200 to achieve a 5 μM concentration and Hoechst (Thermo Fisher, USA) diluted 1:1000 to reach a 1 μg/mL concentration. Both were added to 3 mL of DPBS (Sigma‒Aldrich, USA). Next, the culture medium surrounding the sIOs in the imaging dish was gently removed, and the sIOs were rinsed with DPBS. Next, a staining solution was applied to the sIOs to ensure uniform coverage. The organoids were then incubated for 1.5 hours to ensure optimal staining. After this duration, the excess staining solution was gently aspirated, followed by a final rinse with DPBS to remove any residual dye.

### Data acquisition

The center of the Matrigel dome was found manually with the help of a brightfield preview image scanned over a 4 mm × 4 mm area. Randomly selected organoids within the preview image were captured with a field of view (FOV) of 160 μm × 160 μm and a depth range of up to 140 μm; measuring an axial stack of each FOV took less than 10 seconds. For organoids exceeding the size of the FOV, multiple holotomograms were acquired to construct a stitched volume. The stitching process was automatically performed via the software TomoStudio X (Tomocube Inc., Korea).

To horizontally stitch multiple FOVs together, a 15-µm overlap was used during data acquisition. To ensure accurate stitching under the limited precision of the motorized stage, we computationally calibrated the horizontal coordinates after measurement. For each pair of adjacent FOVs, the *x* and *y* coordinate offsets were adjusted to maximize the Pearson correlation in the overlapping regions^26^. This process was repeated for all the overlapping regions. Finally, pyramid blending^27^ was used to seamlessly merge the tiles.

For time-lapse imaging, humidity was maintained by replenishing the incubation chamber with 5 mL/day deionized H_2_O. The temperature and CO_2_ concentration were automatically adjusted to 37 °C and 5%, respectively, by the chamber controller unit.

### Image processing for segmentation

Each stack of raw holotomograms was processed with MATLAB (MathWorks, USA). To segment the organoids of interest, we calculated a gradient image from the raw image by applying a Sobel filter to each XY-slice. Stacks were cropped along the z-axis within a range determined by the thresholding mean intensity of the gradient, which represents the in-focus features in each slice. After applying this filter via MATLAB, we segmented the organoids across several slices. Using ilastik^28^, we inferred organoid-occupied regions based on the feature distribution of the manual segmentation. Finally, we manually examined the inferred segmentation and trimmed it if necessary due to misinference.

To visualize 3D structures in 2D figures, gradient image stacks were depth color-coded and projected onto a single plane via maximum intensity projection. The applied color map is indicated by a color bar in each produced image.

### Feature extraction

After the organoid mask was obtained through the segmentation process, it was processed in MATLAB to determine the volume through the built-in function ‘regionprops3’. The number of voxels was converted to volume by adjusting for both lateral and axial resolution. Protein density was derived from the organoid’s RI, with an increment value of 0.185 ng/μm used in this study. The protein mass of the organoids was then calculated by multiplying volume by protein density. Histograms of the organoids were generated with the built-in MATLAB function ‘histogram’. Regions of interest in the histograms were specifically designated using the organoid mask to exclude non-organoid regions.

### Statistical analysis

Statistical analyses were performed in MATLAB using a two-tailed, unpaired Student’s t test. A *p* value of < 0.05 was considered statistically significant, and the data are presented as means ± s.e.m.

## Results

### Low-coherence HT reveals distinct structures of native sIOs

**Fig. 1:**
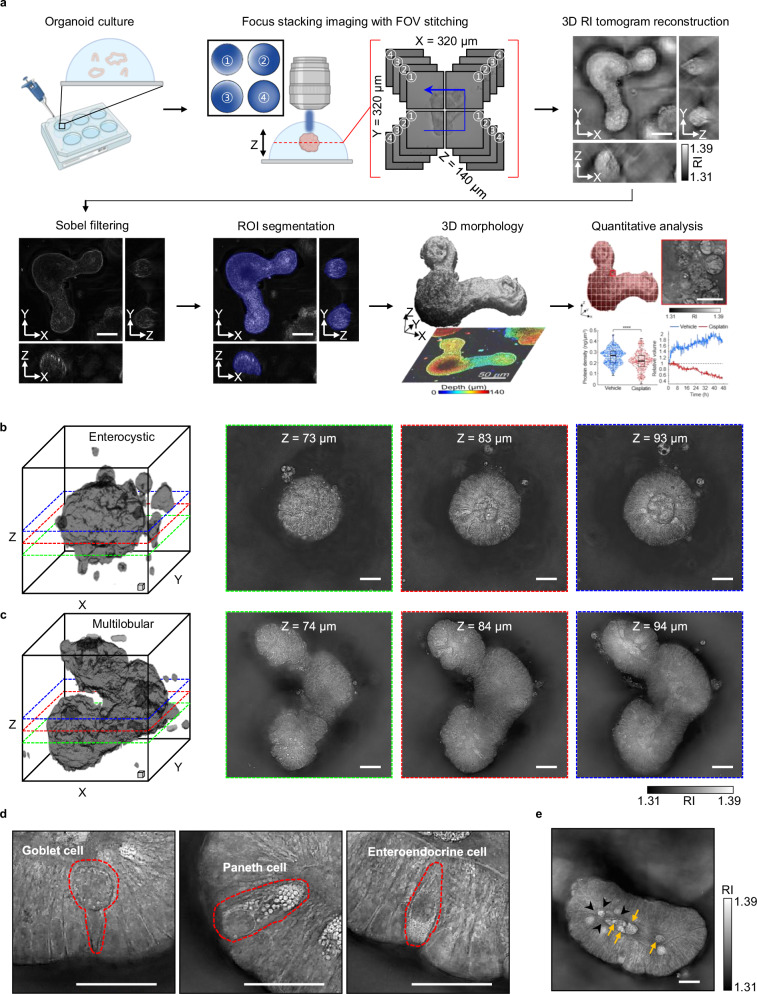
High-resolution holotomograms of live mouse sIOs exhibiting detailed structures. **a** Overview of the low-coherence HT workflow. **b**, **c** Representative holotomograms of sIOs displaying two distinct morphologies: enterocystic organoids (b) and multilobular organoids (**c**). The 3D rendered images are optically sectioned within a cubic box, with each axial slice on the right-hand side delineated by colored dashed boxes. Scale boxes represent a volume of 10 µm × 10 µm × 10 µm (width, height, and depth, respectively). **d** Detailed subcellular-level structures. The red dotted lines represent cell boundaries. **e** Macroscopic structures revealed in stitched sIO holotomograms. The black arrowheads represent the brush borders of the sIOs. The orange arrows indicate exfoliated cells and debris from dead cells. All the scale bars in this figure represent 20 µm.

To comprehensively examine and continuously observe live sIOs, we developed protocols for initial steps such as subculturing, followed by imaging, segmentation, and detailed morphological and quantitative analyses (Fig. 1a). The first step involves splitting mature sIOs and subculturing these fragments within Matrigel domes that mimic the extracellular matrix, targeting 5–10 organoids per dome. Following a five-day incubation, the organoid samples are relocated to coverslip-bottom dishes for imaging. Low-coherence HT is then employed using four distinct patterned beams to illuminate the samples^24^. These raw images undergo a deconvolution algorithm for reconstruction. As the organoids grow beyond the lateral field of view (160 µm × 160 µm) of the imaging device, tiled image capture becomes necessary, followed by post-acquisition stitching. The reconstructed images show the distribution of the refractive index (RI) within the sIOs. When multiple organoids are present in a volume and out-of-focus beams interfere with illumination, sample-induced aberrations are inevitable. To counteract such aberration-induced artifacts, which obscure organoid boundaries, a Sobel filter is applied. This results in synthetically generated images with clearly delineated boundaries. Further segmentation and labeling of organoid outlines are accomplished via the open-source user-interactive segmentation toolkit ilastik^28^. To track the axial growth of sIOs, color coding along the axial direction is employed, with images projected in a consistent orientation. Within these segmented images, parameters such as volume, protein concentration, and mass are quantitatively assessed, enabling comprehensive, longitudinal tracking of organoid features.

With our 3D reconstructed images of sIOs, we identified two types of sIOs on the basis of their morphology: enterocystic sIOs (Fig. 1b) and multilobular sIOs (Fig. 1c). At the early stages of organoid growth, enterocystic sIOs were commonly observed, whereas at the later stages, the emergence of multilobular structures prevailed, primarily due to crypt budding. These observations are consistent with previous studies on long-term imaging of sIO growth^29,30^. Through optical sectioning of reconstructed HT images, we elucidated the complex internal architecture of sIOs, including goblet cells, Paneth cells, and enteroendocrine cells, each of which exhibited typical morphologies consistent with previously reported electron microscopic studies^31,32^ (Fig. 1d). Additionally, observations of cellular polarity provided valuable insights into the apical lumen and basal environments. Wide-field stitched images further revealed macroscopic features such as brush border formation and the accumulation of exfoliated cells within the apical lumen (Fig. 1e).

### Low-coherence HT captures rapid dynamic changes in sIOs

**Fig. 2:**
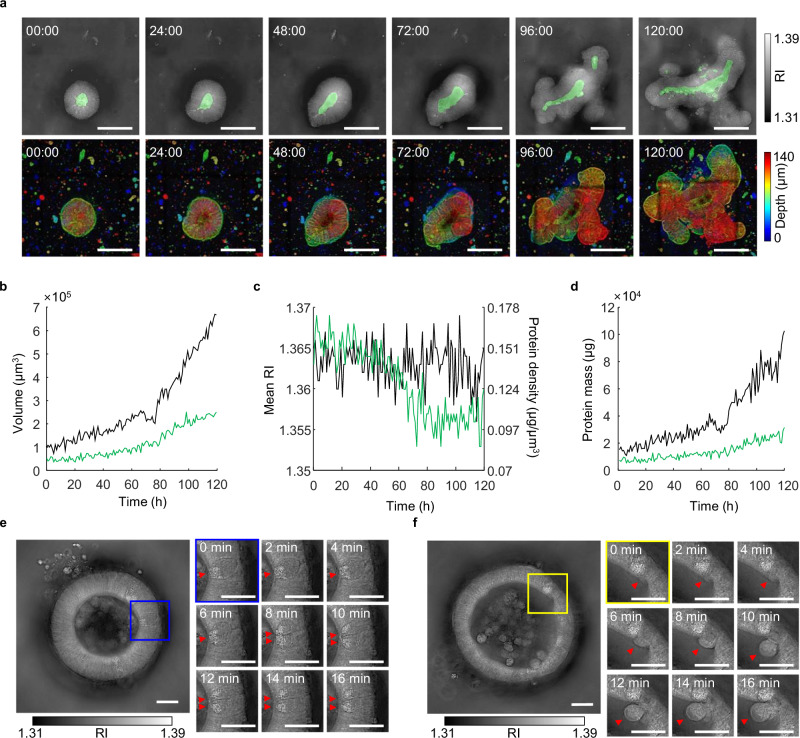
Dynamic changes in live sIOs captured through low-coherence HTs. **a** Sequential images depicting sIO development. At each time point (hour:minute), focal plane images are shown in the upper row, whereas depth-color-coded projections appear in the lower row. In the upper row images, the lumen is segmented and highlighted in green. Scale bar = 50 µm. **b**–**d** Graphical illustration of sIO size (**b**), mean RI and protein density (**c**), and protein dry mass (**d**) over time. The black line represents the entire sIO, whereas the green line specifically denotes the lumen. **e**, **f** Time-lapse images of cellular mitosis (**e**) and exfoliation (**f**). At each timeframe, the z-position was adjusted to the best focal plane for the targeted cells. Scale bar = 20 µm.

To systematically examine sIO development, Matrigel-embedded organoids were subjected to hourly imaging over a span of 120 hours. At the initial enterocystic stage, we noted phenomena such as symmetry breaking and protrusion, eventually leading to the budding of crypts (Fig. 2a). The axial progression of cyst formation and crypt budding was vividly captured via projected depth-color-coded images. By applying segmented masks to Sobel-filtered images, we were able to track organoid size over the entire imaging period, revealing an exponential growth trend (Fig. 2b).

Furthermore, we segmented and tracked the volume of the lumens within the sIOs, documenting an increase in volume corresponding with organoid growth. Given the linear relationship between RI and protein density in biological samples^33,34^, we calculated the protein density in the sIOs (Fig. 2c). Our findings indicate that the total protein concentration in the sIOs remained relatively constant in comparison with that in the luminal space, suggesting there were minimal changes in compositional makeup. As the sIOs increased in size, we noted a reduction in lumen volume, likely due to diluted protein density caused by the increased presence of empty space from exfoliated cells within the lumen. By integrating volume and protein density measurements, we successfully determined the dry protein mass of the sIOs and their luminal contents (Fig. 2d).

Owing to the rapid acquisition capabilities of low-coherence HTs, we were able to capture the dynamic behavior of live sIOs embedded in Matrigel. While specific dividing cell types were not identified, key mitotic events, including apical cytokinesis and cell interspersion, followed by basal reattachment, were observed (Fig. 2e). These observations correlate well with established mitotic features from prior studies^35^. Moreover, we documented epithelial cell exfoliation and subsequent cellular apoptosis and chromatin condensation (Fig. 2f). While previous studies were limited to observing intestinal cell exfoliation^36,37^, our study captures the entire process, including the intercellular migration and apoptosis of exfoliated cells, supporting the long-debated notion that exfoliation removes senescent or damaged enterocytes^38^.

### Observation of drug response in organoids with low-coherence HT

**Fig. 3:**
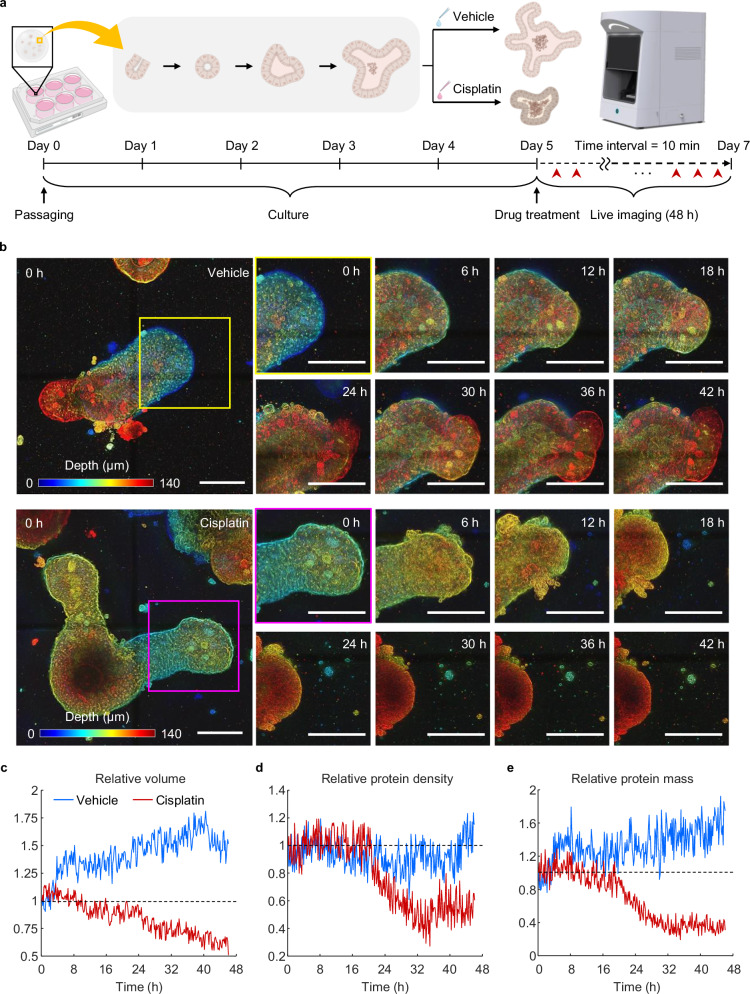
Quantitative evaluation of drug responsiveness in sIOs via low-coherence HTs. **a** Diagram illustrating holotomogram-based viability assessment of drug-treated sIOs. The red arrowheads highlight the time points at which the images were captured. **b** Time-lapse images of sIOs following drug treatment. Each colored box is magnified, with the color bar indicating sample depth. Scale bar = 20 µm. **c**–**e** Longitudinal quantitative assessment of volume (**c**), protein density (**d**), and protein mass (**e**). Every parameter is calibrated against the initial measurement; the dotted line signifies the baseline value.

To evaluate cell death and viability within the organoids, sIOs were treated with cisplatin, and morphological changes were closely observed. We cultured the sIOs in media containing varying concentrations of cisplatin to determine the optimal concentration for discernible toxic effects. We identified the optimal concentration as 10 μM, which was corroborated via brightfield microscopy. Dimethyl sulfoxide (DMSO) served as a vehicle and positive control. Post-treatment, the samples were placed in the imaging system incubator and imaged at 10-minute intervals over a 48-hour span (Fig. 3a). Post-cisplatin, we observed that the branched crypts underwent noticeable shrinkage and displayed an increasing number of dissociated dead cells. In contrast, the vehicle-treated organoids exhibited enhanced crypt budding and growth (Fig. 3b). The observed reduction in organoid volume can likely be attributed to cellular shrinkage and the exfoliation and dissociation of dead cells, as previously reported^39^. Quantitative tracking of organoid size further supports these observations (Fig. 3c). While the volume of vehicle-treated sIOs increased, that of those treated with cisplatin decreased. The protein density in the vehicle-treated group remained constant, whereas a significant decrease was observed in the cisplatin-treated group after 24 hours (Fig. 3d). The overall protein mass was increased in the vehicle-treated sIOs, whereas it was markedly decreased in the cisplatin-treated group (Fig. 3e).

### Quantification of viability via low-coherence HTs reveals a decrease in RI

**Fig. 4:**
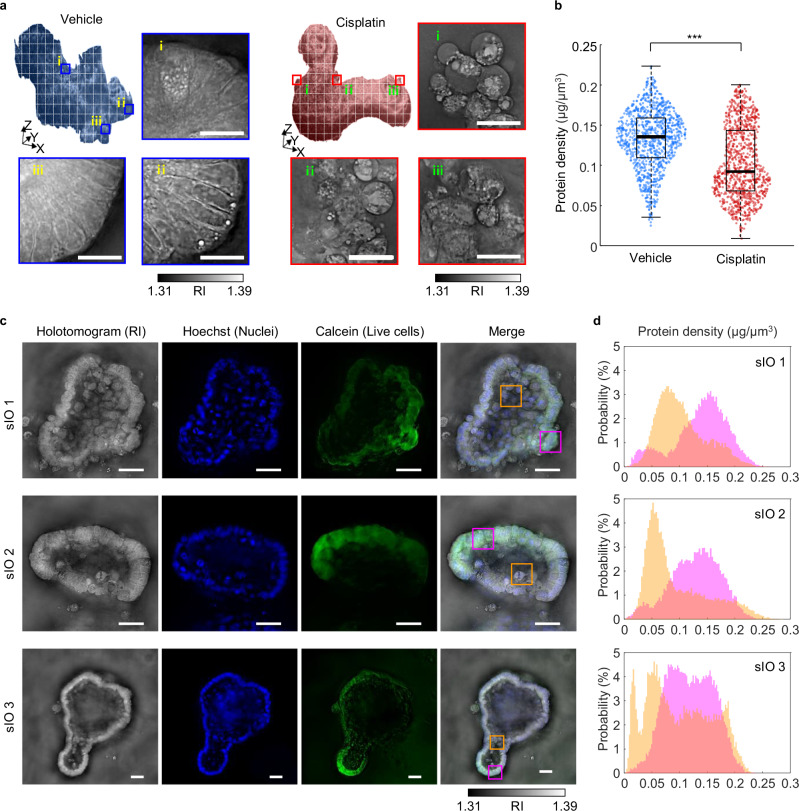
In-depth quantitative analysis of sIO viability. **a** Illustration of the subvolume sectioning strategy accompanied by representative images. Scale bar = 20 µm. **b** Scatter plot of the protein density of vehicle- and cisplatin-treated sIOs. Each dot represents a subvolume of sectioned images (*N* = 1053 for vehicle, *N* = 1005 for cisplatin). The bottom and top of each box signify the 25th and 75th percentiles of the subvolumes, respectively. The distance between the bottom and top of each box gives the interquartile range, and the bold line in the middle of each box indicates the median. The t test results are as indicated; ***p < 0.001. **c** Correlative images of sIOs labeled with Hoechst (blue) and calcein-AM (green). In the merged images, the magenta and orange boxes indicate live cell-dominant and dead cell-dominant regions, respectively. Scale bar = 50 µm. **d** Histograms illustrating the protein density distribution within the boxed regions of (**c**). Each color corresponds to the color of the box.

To further elucidate the marked decrease in protein density observed in the cisplatin-treated group, we investigated the RI distribution within the sIOs. Initially, our aim was to perform a cell-by-cell analysis by segmenting individual cells with single-cell masks. However, this approach was impeded by indistinct cell boundaries and cell clumping within the organoids. To circumvent this challenge, we partitioned the holotomograms of the sIOs into multiple cubic sections, each measuring 50 μm × 50 μm laterally and 10 μm axially. The cubic size was set large enough to cover a single cell, ensuring that each cubic section contained an entire single enterocyte for more accurate analysis (Fig. 4a). We compared the protein density of each cubic section of the organoids of cisplatin-treated sIOs and those of vehicle-treated sIOs after 24 hours of treatment (Fig. 4b). The cisplatin-treated group had a lower protein density (mean ± std; 0.104 ± 0.045 ng/μm^3^) than the vehicle-treated group (mean ± std; 0.133 ± 0.037 ng/μm^3^). We hypothesize that the lower protein density in the cisplatin-treated group originated from the increased number of dead cells in the cisplatin-treated sIOs because the dead cell region exhibited a low RI (Fig. 4a). Previous studies have reported that, during cell death, a decrease in the phase of scattered beams from cells also indicates a decrease in RI, which supports our hypothesis^40^. To validate our hypothesis, we stained sIOs with Hoechst and calcein AM and obtained fluorescence images of low-coherence HTs (Fig. 4c). Since Hoechst stains the whole nucleus of cells in any live/dead state while calcein-AM stains only live cells, we can identify the live-cell dominant and dead-cell dominant regions in the images. Notably, in intestinal organoids, apoptotic cells are often expelled into the lumen^41^, resulting in the lumen solely exhibiting Hoechst signals. We compared the protein density of the co-stained (live-cell dominant) and Hoechst-only stained (dead-cell dominant) regions. Histograms of protein density revealed different distributions for the costained and Hoechst-only regions (Fig. 4d). Compared with the live-cell-dominant regions (mean ± std; 0.115 ± 0.050 ng/μm^3^), the dead-cell-dominant regions had lower protein density (mean ± std; 0.101 ± 0.048 ng/μm^3^).

## Discussion

In summary, low-coherence HT is a real-time, label-free method for organoid imaging, enabling researchers to explore the detailed complexities of organoids without time-consuming preparation and labeling. Using low-coherence HT, we were able to capture long-term, real-time observations of biological processes within organoids, such as cell apoptosis, migration, mitosis, and subcellular organelle dynamics, at a resolution between brightfield and electron microscopy. High-resolution 3D observations are difficult to achieve in real time via conventional imaging methods, particularly in intact organoids in Matrigel media. This study presents advancements in two primary domains. First, specific sample preparation steps, including fixation and staining, are required to preserve the structural integrity of live organoids. Second, the quantitative analytical capabilities of low-coherence HT provide robust assess to organoid biology, including RI values, making our imaging system a new tool for pharmacological screening applications.

Despite the advantages of using low-coherence HT for organoid imaging, challenges arise when organoids exceed an axial size of approximately 200 µm. Although the lateral coverage range can exceed 1 mm by stitching multiple FOVs, current limitations in the working distance of the objective lens constrain the axial depth coverage. This results in diminished image quality for organoids positioned in the upper portion of the Matrigel dome, far from the objective lens. We note that hardware improvements such as long-working-distance objective lenses effectively reduce this aberration issue albeit with additional hardware cost.

In addition, intense light scattering by organoids can introduce sample-induced aberrations, further compromising image quality. Since our organoid samples are embedded in spherical Matrigel domes, they are prone to spherical aberrations. Additionally, multiple scattering events stemming from the thickness of organoids contributes to further aberrations, which we refer to as sample-induced aberrations. To address these aberrations, various advanced methods can be applied, including adaptive optics techniques such as deformable mirrors and spatial light modulators, which correct wave front distortions in real time^42^. Computational algorithms can also be used to correct phase distortions in captured holograms post-acquisition.

Recently, a method based on partial reconstruction and wave-backpropagation techniques was applied to numerically address multiple scattering and sample-induced aberrations^43^. Although it cannot be directly applied to our study owing to the difference between coherent HTs and partially coherent HTs, we anticipate that future computational improvements in HT reconstruction will provide more detailed insights into organoids. In addition to physically modeled reconstruction methods, aberrations can potentially be corrected via a convolutional neural network (CNN) trained with phase images, handling aberration compensation as a regression task to predict optimal coefficients for constructing phase aberration maps^44^.

In our current imaging system, we must address the challenge of low biomolecular specificity. A potential solution for overcoming this limitation is through the implementation of correlative imaging techniques, which can facilitate the differentiation of individual subcellular structures. As recently demonstrated, deep learning algorithms can accurately predict subcellular structures based on distinct features in the spatial distribution of the RI associated with each structure^45–47^. Such predictions can be applied to label-free time-lapse imaging of unlabeled live organoids. We expect that combining correlative imaging with artificial intelligence could lead to improved biomolecular specificity in our imaging system. Furthermore, validation of this approach using labeling agents targeting specific molecules would generate additional confidence in its effectiveness.

Interestingly, as shown in Fig. 4d, regions stained with both calcein and Hoechst (live regions, magenta box) have a higher RI than regions stained only with Hoechst (dead regions, orange box). Since RI is linearly proportional to protein density^33,34^, regions with apoptotic or dead cells degraded into small debris by caspases^48^ have low protein concentration and consequently low RI values. This is observed in Fig. 2c, which shows the intestinal lumen’s low mean RI over time. As apoptotic cells exfoliate into the lumen, a decrease in mean RI in the lumen is consistent with our results. Therefore, HT enables live/dead assays without exogenous agents, allowing for the quantification of organoid viability.

The large size of volumetric organoid holotomograms, approximately 100 GB per organoid, is associated with high processing times, posing a significant challenge for the proposed method. The emergence of high-throughput platforms equipped with advanced computing power will enable the analysis of intricate cellular morphologies and dynamic changes within multiple organoids at high resolution. These technological advancements promise to revolutionize the field of regenerative medicine, offering a robust means of validating the quality of organoids destined for therapeutic applications. The present study provides evidence for the application of low-coherence HT as an organoid imaging modality that could prompt a shift in drug screening paradigms, potentially leading to more personalized and efficient treatment strategies.

## References

[1] Clevers, H. Modeling development and disease with organoids. *Cell* **165**, 1586–1597 (2016). 27315476 10.1016/j.cell.2016.05.082

[2] Fatehullah, A., Tan, S. H. & Barker, N. Organoids as an in vitro model of human development and disease. *Nat. Cell Biol.* **18**, 246–254 (2016). 26911908 10.1038/ncb3312

[3] Kim, J., Koo, B.-K. & Knoblich, J. A. Human organoids: model systems for human biology and medicine. *Nat. Rev. Mol. Cell Biol.* **21**, 571–584 (2020). 32636524 10.1038/s41580-020-0259-3 PMC7339799

[4] Sato, T. et al. Long-term expansion of epithelial organoids from human colon, adenoma, adenocarcinoma, and Barrett’s epithelium. *Gastroenterology* **141**, 1762–1772 (2011). 21889923 10.1053/j.gastro.2011.07.050

[5] Yui, S. et al. Functional engraftment of colon epithelium expanded in vitro from a single adult Lgr5+ stem cell. *Nat. Med.* **18**, 618–623 (2012). 22406745 10.1038/nm.2695

[6] Kim, M. et al. Patient-derived lung cancer organoids as in vitro cancer models for therapeutic screening. *Nat. Commun.* **10**, 3991 (2019). 31488816 10.1038/s41467-019-11867-6 PMC6728380

[7] Dye, B. R. et al. In vitro generation of human pluripotent stem cell derived lung organoids. *elife* **4**, e05098 (2015). 25803487 10.7554/eLife.05098 PMC4370217

[8] Huch, M. et al. In vitro expansion of single Lgr5+ liver stem cells induced by Wnt-driven regeneration. *Nature* **494**, 247–250 (2013). 23354049 10.1038/nature11826 PMC3634804

[9] Huch, M. et al. Long-term culture of genome-stable bipotent stem cells from adult human liver. *Cell* **160**, 299–312 (2015). 25533785 10.1016/j.cell.2014.11.050 PMC4313365

[10] Takebe, T. et al. Vascularized and functional human liver from an iPSC-derived organ bud transplant. *Nature* **499**, 481–484 (2013). 23823721 10.1038/nature12271

[11] Takasato, M. et al. Kidney organoids from human iPS cells contain multiple lineages and model human nephrogenesis. *Nature* **526**, 564–568 (2015). 26444236 10.1038/nature15695

[12] Homan, K. A. et al. Flow-enhanced vascularization and maturation of kidney organoids in vitro. *Nat. Methods* **16**, 255–262 (2019). 30742039 10.1038/s41592-019-0325-y PMC6488032

[13] Lancaster, M. A. et al. Cerebral organoids model human brain development and microcephaly. *Nature* **501**, 373–379 (2013). 23995685 10.1038/nature12517 PMC3817409

[14] Quadrato, G. et al. Cell diversity and network dynamics in photosensitive human brain organoids. *Nature* **545**, 48–53 (2017). 28445462 10.1038/nature22047 PMC5659341

[15] Chýlek, P. Absorption and scattering of light by small particles. By C. F. Bohren and d. R. Huffman. *Appl Opt.* **25**, 3166 (1986). 18235596

[16] Fei, K., Zhang, J., Yuan, J. & Xiao, P. Present application and perspectives of organoid imaging technology. *Bioengineering* **9**, 121 (2022). 35324810 10.3390/bioengineering9030121 PMC8945799

[17] Keshara, R., Kim, Y. H. & Grapin-Botton, A. Organoid imaging: seeing development and function. *Annu. Rev. Cell Dev. Biol.* **38**, 447–466 (2022). 35767871 10.1146/annurev-cellbio-120320-035146

[18] Rios, A. C. & Clevers, H. Imaging organoids: a bright future ahead. *Nat. methods* **15**, 24–26 (2018). 29298292 10.1038/nmeth.4537

[19] Park, Y., Depeursinge, C. & Popescu, G. Quantitative phase imaging in biomedicine. *Nat. Photonics* **12**, 578–589 (2018).

[20] Pirone, D. et al. Stain-free identification of cell nuclei using tomographic phase microscopy in flow cytometry. *Nat. photonics* **16**, 851–859 (2022). 36451849 10.1038/s41566-022-01096-7 PMC7613862

[21] Jin, D., Zhou, R., Yaqoob, Z. & So, P. T. Tomographic phase microscopy: principles and applications in bioimaging. *JOSA B* **34**, B64–B77 (2017). 29386746 10.1364/josab.34.000b64 PMC5788179

[22] Wu, Y. et al. Intelligent frequency-shifted optofluidic time-stretch quantitative phase imaging. *Opt. Express* **28**, 519–532 (2020). 32118978 10.1364/OE.380679

[23] Liu, Y. & Uttam, S. Perspective on quantitative phase imaging to improve precision cancer medicine. *J. Biomed. Opt.* **29**, S22705–S22705 (2024). 38584967 10.1117/1.JBO.29.S2.S22705 PMC10996848

[24] Hugonnet, H., Lee, M. & Park, Y. Optimizing illumination in three-dimensional deconvolution microscopy for accurate refractive index tomography. *Opt. Express* **29**, 6293–6301 (2021). 33726154 10.1364/OE.412510

[25] Hugonnet, H., Han, H., Park, W. & Park, Y. Improving specificity and axial spatial resolution of refractive index imaging by exploiting uncorrelated subcellular dynamics. *ACS Photonics* **11**, 257–266 (2023).

[26] Hugonnet, H. et al. Multiscale label-free volumetric holographic histopathology of thick-tissue slides with subcellular resolution. *Adv. Photonics* **3**, 026004–026004 (2021).

[27] Burt, P. J. & Adelson, E. H. A multiresolution spline with application to image mosaics. *ACM Trans. Graph. (TOG)* **2**, 217–236 (1983).

[28] Berg, S. et al. ilastik: interactive machine learning for (bio)image analysis. *Nat. Methods* **16**, 1226–1232 (2019). 31570887 10.1038/s41592-019-0582-9

[29] Sato, T. et al. Single Lgr5 stem cells build crypt-villus structures in vitro without a mesenchymal niche. *Nature* **459**, 262–265 (2009). 19329995 10.1038/nature07935

[30] de Medeiros, G. et al. Multiscale light-sheet organoid imaging framework. *Nat. Commun.* **13**, 4864 (2022). 35982061 10.1038/s41467-022-32465-z PMC9388485

[31] Miura, S. & Suzuki, A. Generation of Mouse and Human Organoid-Forming Intestinal Progenitor Cells by Direct Lineage Reprogramming. *Cell Stem Cell* **21**, 456–471.e455 (2017). 28943029 10.1016/j.stem.2017.08.020

[32] Dicarlo, M. et al. Quercetin Exposure Suppresses the Inflammatory Pathway in Intestinal Organoids from Winnie Mice. *Int. J. Mol. Sci.* **20**, 5771 (2019). 31744123 10.3390/ijms20225771 PMC6888448

[33] Barer, R. Determination of dry mass, thickness, solid and water concentration in living cells. *Nature* **172**, 1097–1098 (1953). 13111263 10.1038/1721097a0

[34] Popescu, G. et al. Optical imaging of cell mass and growth dynamics. *Am. J. Physiol.-Cell Physiol.* **295**, C538–C544 (2008). 18562484 10.1152/ajpcell.00121.2008 PMC2518415

[35] McKinley, K. L. et al. Cellular aspect ratio and cell division mechanics underlie the patterning of cell progeny in diverse mammalian epithelia. *eLife* **7**, e36739 (2018). 29897330 10.7554/eLife.36739 PMC6023609

[36] Kaeffer, B. et al. Recovery of Exfoliated Cells From the Gastrointestinal Tract of Premature Infants: A New Tool to Perform “Noninvasive Biopsies?”. *Pediatr. Res.* **62**, 564–569 (2007). 17805197 10.1203/PDR.0b013e318155a402

[37] Ahlquist, D. A., Harrington, J. J., Burgart, L. J. & Roche, P. C. Morphometric analysis of the “mucocellular layer” overlying colorectal cancer and normal mucosa: Relevance to exfoliation and stool screening. *Hum. Pathol.* **31**, 51–57 (2000). 10665913 10.1016/s0046-8177(00)80198-7

[38] Loktionov, A. Cell exfoliation in the human colon: Myth, reality and implications for colorectal cancer screening. *Int. J. Cancer* **120**, 2281–2289 (2007). 17351899 10.1002/ijc.22647

[39] Grabinger, T. et al. Ex vivo culture of intestinal crypt organoids as a model system for assessing cell death induction in intestinal epithelial cells and enteropathy. *Cell Death Dis.* **5**, e1228–e1228 (2014). 24832600 10.1038/cddis.2014.183 PMC4047863

[40] Pavillon, N. et al. Early Cell Death Detection with Digital Holographic Microscopy. *PLoS One* **7**, e30912 (2012). 22303471 10.1371/journal.pone.0030912 PMC3269420

[41] Bullen, T. F. et al. Characterization of epithelial cell shedding from human small intestine. *Lab. Investig.* **86**, 1052–1063 (2006). 16909128 10.1038/labinvest.3700464

[42] Sirico, D. G. et al. Compensation of aberrations in holographic microscopes: main strategies and applications. *Appl. Phys. B* **128**, 78 (2022).

[43] Yasuhiko, O. & Takeuchi, K. In-silico clearing approach for deep refractive index tomography by partial reconstruction and wave-backpropagation. *Light.: Sci. Appl.* **12**, 101 (2023). 37105955 10.1038/s41377-023-01144-z PMC10140380

[44] Xiao, W. et al. Sensing morphogenesis of bone cells under microfluidic shear stress by holographic microscopy and automatic aberration compensation with deep learning. *Lab a Chip* **21**, 1385–1394 (2021). 10.1039/d0lc01113d 33585849

[45] Jo, Y. et al. Label-free multiplexed microtomography of endogenous subcellular dynamics using generalizable deep learning. *Nat. Cell Biol.* **23**, 1329–1337 (2021). 34876684 10.1038/s41556-021-00802-x

[46] Bai, B. et al. Deep learning-enabled virtual histological staining of biological samples. *Light.: Sci. Appl.* **12**, 57 (2023). 36864032 10.1038/s41377-023-01104-7 PMC9981740

[47] Park, J. et al. Artificial intelligence-enabled quantitative phase imaging methods for life sciences. *Nat. Methods* **20**, 1645–1660 (2023). 37872244 10.1038/s41592-023-02041-4

[48] Taylor, R. C., Cullen, S. P. & Martin, S. J. Apoptosis: controlled demolition at the cellular level. *Nat. Rev. Mol. Cell Biol.* **9**, 231–241 (2008). 18073771 10.1038/nrm2312

